# Gut Microbiota to Microglia: Microbiome Influences Neurodevelopment in the CNS

**DOI:** 10.3390/children10111767

**Published:** 2023-10-31

**Authors:** Jeffery Bettag, Daniel Goldenberg, Jasmine Carter, Sylvia Morfin, Alison Borsotti, James Fox, Matthew ReVeal, Dylan Natrop, David Gosser, Sree Kolli, Ajay K. Jain

**Affiliations:** 1 Department of Pediatrics, Saint Louis University School of Medicine, Saint Louis, MO 63103, USA; daniel.goldenberg@health.slu.edu (D.G.); jasmine.carter.1@health.slu.edu (J.C.); sylvia.e.morfin@slu.edu (S.M.); alison.borsotti@health.slu.edu (A.B.); james.fox@health.slu.edu (J.F.); matthew.reveal@health.slu.edu (M.R.); david.gosser@slu.edu (D.G.); sree.kolli@slu.edu (S.K.); ajay.jain@slucare.ssmhealth.com (A.K.J.); 2 Medical College of Wisconsin-Green Bay, De Pere, WI 54115, USA; dnatrop@mcw.edu

**Keywords:** microglia, gut microbiome, Gut–Brain axis, metabolite, neurodevelopment

## Abstract

The brain is traditionally viewed as an immunologically privileged site; however, there are known to be multiple resident immune cells that influence the CNS environment and are reactive to extra-CNS signaling. Microglia are an important component of this system, which influences early neurodevelopment in addition to modulating inflammation and regenerative responses to injury and infection. Microglia are influenced by gut microbiome-derived metabolites, both as part of their normal function and potentially in pathological patterns that may induce neurodevelopmental disabilities or behavioral changes. This review aims to summarize the mounting evidence indicating that, not only is the Gut–Brain axis mediated by metabolites and microglia throughout an organism’s lifetime, but it is also influenced prenatally by maternal microbiome and diet, which holds implications for both early neuropathology and neurodevelopment.

## 1. Introduction

The gastrointestinal tract houses a diverse community of trillions of bacteria that exist in a symbiotic relationship with their host organism. These commensal bacteria influence the host through facilitation of digestion, immune system modulation, and influencing neurologic function and behavior [1,2,3]. Additionally, the gut microbiome has been demonstrated to be vital for the establishment of the blood–brain barrier (BBB), as germ-free (GF) mice were found to have brain vascular endothelium that were highly permeable to macromolecules and recovered selective permeability upon colonization with non-pathogenic gut microbiome or through exposure to short chain fatty acid metabolites of gut bacteria [4].

Microglia are immune cells native to the brain, which have an important role in modulating inflammation in the brain, neurogenesis, and synaptic architecture. These cells are the most abundant immune-related cells in the brain [5]. The brain is considered a relatively immunologically privileged site due to lack of lymphatic drainage and its reduced ability to present antigens to induce immune responses. However, the central nervous system (CNS) [6] does possess an array of unique cells, associated with the innate immune system, that serve a central role in mounting immune responses within the CNS [6]. Microglial cells, specifically, are prevalent in brain parenchyma. Augmentation or inhibition of immune responses have known clinical impact in controlling a host of neurodevelopmental or neurodegenerative diseases. For instance, it has been shown that SGLT2 inhibitors can reduce neuroinflammation and be neuroprotective by downregulating microglia that have been activated by bacterial factors; however, these studies were on adults who are well beyond early neurodevelopment [7]. It is becoming increasingly known that gut bacteria release certain factors similar to those seen in acute inflammatory states. Therefore, the modulation of these bacteria, the control of the factors released, the progressive development of the microbiome, and its associated bacteria and factors are likely to have an important role to play in early childhood neurodevelopment. This is becoming an increasingly important field, as, in children, who have not achieved full neurodevelopment and therefore are more prone to neurological insult, the microbiome may shape cognitive outcomes, impacting psychiatric illnesses, overall morbidity, and other behavioral factors. The aim of this literature review is to dissect the embryological development of microglia in the context of maternal and prenatal microbiomes and their overall impact on global neurodevelopment and associated conditions via Gut–Brain signaling.

## 2. Embryologic Characteristics and Development of Microglial Cells

**Figure 1:**
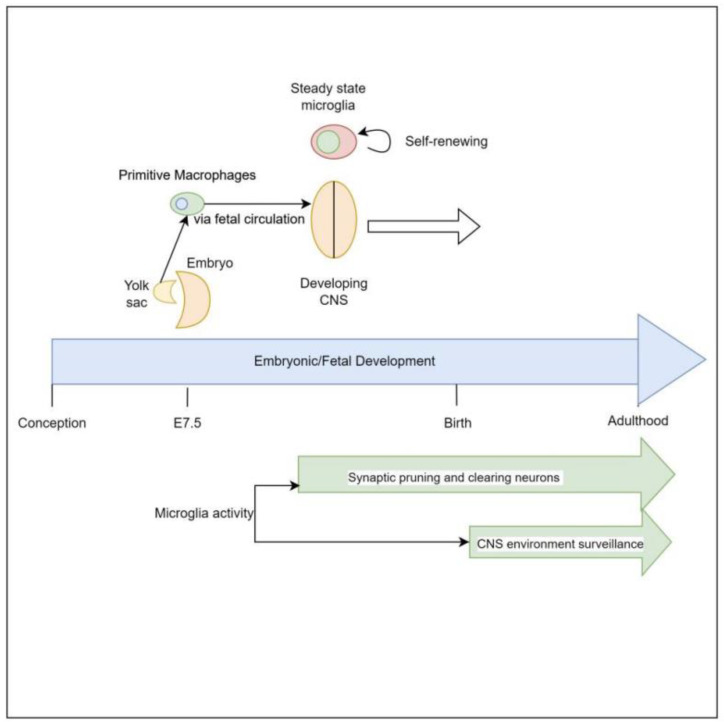
Both in vivo and in vitro, microglia appear to be dependent on Interleukin-34 (IL-34) for stimulation to proliferate. IL-34 is a cytokine that serves as one ligand to the receptor CSF-1R, which is central to signaling for proliferation and development of mononuclear phagocyte cells [11]. This cytokine is strongly expressed in mouse brains during embryogenesis, and strongly correlates with the proliferation of mononuclear phagocytic cells [12,13]. Additionally, IL-34 induces microglia to produce insulin degrading enzyme (IDE) and heme oxygenase-1 (HO-1), which are associated with clearing oligomeric amyloid Beta and defend against reactive oxygen species, respectively [14].

Microglia are derived from primitive myeloid progenitor cells originating from the yolk sack on embryonic day 7.5 (E7.5) via stimulation by colony stimulating factor 1 receptor (CSF-1R). These primitive cells penetrate the CNS via pial surfaces and the fourth ventricle [8]. This represents a developmental path distinct from that of the hematopoietic macrophage cells present in the rest of the body such as red pulp macrophages, subcapsular sinus macrophages, alveolar macrophages of the lungs, and Kupffer cells of the liver. Microglial cells are maintained throughout the organism’s lifespan, independent of hematopoietically derived macrophages [8,9]. Evidence supporting this yolk sack origin, as distinct from other macrophage lineages, is that 30% of primitive yolk sac macrophages can be labeled early in embryonic development when exposed to tamoxifen, and when the organism is assayed for these labeled cells at different points of embryonic, fetal, and newborn development it reveals consistently a 30% proportion of labeled microglial cells in brain [10] (Figure 1).

## 3. The Effect of Microglial Cells on Neurodevelopment

It is understood that the early brain possesses an abundance of neurons with a plethora of unneeded synaptic connections, which are progressively pruned via early sensory stimulation and motor activity to establish a network that is functional and efficient. This culling and shaping of neuronal connections takes place in designated areas of the brain in accordance with various critical periods—times in which specific stimuli are required [15,16]. This ensures both appropriate physiologic and neurologic development and aids in meeting developmental milestones. For example, in the visual system, if kittens’ eyes are sutured shut in the early months of life there is a marked drop in the number of neuronal cells that are stimulated by those eyes. This study revealed that 6 days of suturing the eyes was equivalent to a 3–4-month physiologic monocular setback [17]. This indicates that lack of sensory stimulation is a significant driving factor in neurodevelopment. Given what is known about microglia, this process is likely to be mediated by microglia.

Microglia are a vital actor during these periods, in a region-specific manner, driving maturation of neural architecture. Microglia appear to survey the extracellular medium for stimuli to induce synaptic pruning. These stimuli vary by region of the brain involved and include complement factor 3 in retinal ganglion cells, IL-33 in the reticular thalamic nucleus, and fractalkine (CX3CR1) in the hippocampus [16,18,19]. Thus, as the thalamus is commonly referred to as the ‘relay center of the brain’ and the hippocampus is known for memory formation, any interaction between gut metabolites and neurologic cell-receptor pathways may result in alterations to memory-forming ability, and thought, motor, and sensory-signaling ability as a result of over, under, or aberrant synaptic pruning. 

## 4. Influence of Microglia on Neuropathology

The importance of microglia-driven synaptic pruning is exemplified by the neuropathology observed when it is disrupted. Autism Spectrum Disorder (ASD) refers to a grouping of communication impairments and atypical behaviors [16]. The cause of ASD is incompletely understood at this time but is believed to be multifactorial, with neuroinflammation (and thus innate immune system activation) being thought to play a strong role in the development of ASD. Autopsy of individuals with ASD, in comparison to matched controls, have revealed significantly higher densities of microglial cells in the fronto-insular cortex, visual cortex, dorsolateral prefrontal cortex, and cerebellum, indicating these cells either play a role in the progression of this syndrome or their proliferation is stimulated by a separate driver [20,21,22,23]. In mice that have overexpression of translation initiation factor eIF4E, there is noted increase in microglial density and phagocytic activity, yet reduced motility and synaptic engulfment [24]. This leads these mice to have higher synaptic density and autism-like behaviors, specifically in male mice vs. female mice. 

As previously mentioned, microglia appear to function as “gardeners” of the early brain, pruning little-utilized synapses, but they also continuously monitor the extracellular medium for signs of injury, infection, or cellular derangement to protect the CNS via modulation of inflammation, stimulation of neurogenesis, and clearing of cellular debris [25]. As the microbiome and microglia are both constantly developing from birth to death, maturing the gut, immune system, and neurodevelopment, any interaction between the two can result in such derangements or syndromes. 

## 5. Development of Human Microbiome

The adult microbiome has been immensely studied; it has been found that the average adult microbiome is made up of over 400 species of bacteria [26,27]. An infant’s microbiome is immature and weakened, going through complex development as it progresses through new illnesses or environmental exposures into adulthood. Understanding the development of gut microbiomes is becoming increasingly important in concurrent research into cognitive development. Studies have been conducted that show that an infant’s microbiome goes through exponential development over the course of the first few years of life, eventually leading to an extensive, stable gut by the age of three [26]. Throughout these first three years of life, the infant’s microbiome experiences successive growth with several shifts between different bacterial taxonomies [26]. 

Major factors in the establishment and maintenance of the infant microbiome reside in both maternal and infant factors. Maternal exposures, such as infection, stress, and obesity, play a critical role in the establishment of the microbiome of the neonate after birth. Infant factors, such as mode of delivery, antibiotic exposure, and infant nutritional intake, are additional vital contributors. Mode of delivery is an integral contributor to the establishment of the gut microbiome. Infants who have been born vaginally have their initial gut flora similar to that of their mothers vaginal and anal flora, consisting heavily of *Lactobacillus*, while those born via cesarean section have their initial gut flora closely resembling bacteria heavily colonized on the skin surface, such as *Staphylococcus* [26]. Infants who were born via cesarean section have been found to have less diversity in their intestinal flora, and these changes in the gut microbiome can be seen until the age of seven [26]. Salminen et al. conducted a study in 2004 which assessed differences in intestinal flora of seven-year-old children who were born vaginally versus those who were born by cesarean section; results signified that there are significantly more Clostridium species in those who were born vaginally, signifying that changes in the microbial gut continue beyond delivery [28].

Antibiotic exposure in utero and throughout the formulation of the gut microbiome alters the trajectory and inhabitants of the gut bacteria. Studies conducted by Aloisio et al. in 2014 demonstrated that newborns whose mothers were found to be group B Streptococcus (GBS) positive and received antibiotic prophylaxis had counts of Bifidobacteria that were significantly decreased compared to the control group whose mothers did not receive prophylactic antibiotics for GBS [29]. The use of antibiotics in utero and thereafter leads to alterations in the gut microbiome and a delay in the establishment of commensal flora.

Diet composition from birth and into adulthood has a radical influence on the development of the gut microbiome [30]. Harmsen et al. conducted a study in 2000 which showed infants who were breastfed have a predominance of Bifidobacteria, while those infants who were bottle fed displayed a predominance of E. coli, staphylococcus, and clostridium [31]. There have been cited associations between the infant diet, and establishment of its microbial gut, and the development of conditions such as atopy, inflammatory bowel disease, obesity, and metabolic disorders throughout a person’s lifetime [26,32,33]. The transition from breast milk or formula to solid food leads to major alterations in the gut flora shown by a heavy increase in enterobacteria and enterococci [30]. The increase in enterobacteria and enterococci was more significant within infants who were breastfed. Following the introduction of solid food, the population size and makeup of the gut microbiome more closely resembled that of an adult [30]. After the age of three, when the microbial gut resembles that of an adult, alterations in the intestinal flora directly correlate with that of alterations in the makeup of one’s diet [34].

## 6. Microbiome Influence on Microglia

As noted above, microglial cells may have a myriad of interacting signals, to activate, deactivate, proliferate, or senesce, that are dependent on the specific areas of the brain affected or being affected [16,18,19]; however, microglia may also be influenced by factors outside of the CNS. This external influence may be part of normal neural development or represent pathologic disruption of physiologic microglial activity. In particular, the gut microbiome is an expansive ecosystem consisting of trillions of individual organisms comprising thousands of species of bacteria, each metabolically active and interacting with the host digestive system [35,36]. This diverse microbial community serves its’ host by maturing the intestinal mucosa, facilitating breakdown of enteral macromolecules for ease of absorption, maturation of innate immune cells, and production of a myriad of metabolites that appear to have systemic or organ-specific physiologic effects on the host [37,38,39,40]. Recent studies have demonstrated that gut microbiota does have a role in influencing normal neurodevelopment. Germ-free (GF) mice, which lack normal enteric gut commensal bacteria, have been shown to develop increased motor activity and decreased behavioral indicators of anxiety, with normal behavioral phenotypes being restored following intestinal colonization [41]. 

**Figure 2:**
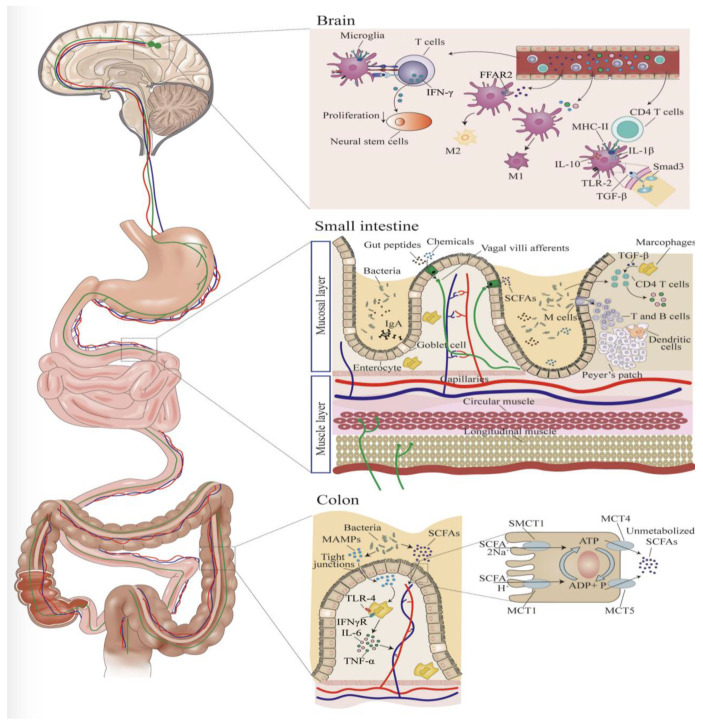
Microbiota influence on microglia. Microbe-associated molecular proteins (MAMPs) are produced by bacteria in the colon. MAMPs travel through tight junctions within the intestinal wall and activate macrophages to stimulate TNF-a and IL-6 release. Metabolites from microbiota, such as short chain fatty acids (SCFAs), are absorbed in the small intestine and begin to interact with T-cells, B-cells, and macrophages, which starts the release of pro-inflammatory cytokines such as TGF-β, IL-6, and TNF-α. Pro-inflammatory cytokines produced in the GI tract will then enter the bloodstream and activate the vagus nerve tract, which carries direct activity to the CNS, directly, or via T-cells that have the capacity to interact with microglia in the brain. CD4 T cells in the brain can interact with microglia and stimulate expression of surface factors, such as MHC-II, IL-10, and IL-2, on microglial cells. TGF-β binds to receptors on microglial cells and downregulates the Smad3 signaling pathway. T-cells can release TNF-α, an inflammatory cytokine after being activated by microglial cells and lead to decreased proliferation of neuronal stem cells, resulting in direct influence on microglial proliferation. “Reproduced with permission from Blackwell Publishing” [47].

In addition to their previously mentioned roles in the development of neuroarchitecture, microglial cells modulate neuroinflammation via pro- and anti-inflammatory chemokines and cytokines [42]. In a study done by Alleva et al. in 1997, it was found that lupus-prone mice had early dysregulation of neuropsychiatric symptoms, such as anhedonia and despair, due to loss of effective TNF-α signaling. This same study linked loss of effective TNF-α production to an increase in dysfunctional cytokines, such as IL-1 and IL-6, which was hypothesized to cause the seen effects [43]. This indicates that, in mice predisposed to immune dysfunction, disruption of cytokine pathways, as is seen in gut dysbiosis, results in altered signaling of the CNS. Once the blood–brain barrier (BBB) was damaged, two separate studies found that IL-1, TNF-α, and IL-6 could diffuse across and cause increased expression of immune cell receptors, such as ICAM-1 and VCAM-1, in the brain parenchyma [44]. Subsequently, this resulted in recruitment of microglia and astrocytes, resulting in cytotoxicity [45,46]. Figure 2 is a diagram of how the gut and brain interact, and exemplifies the expanded pathway by which inflammatory markers have a direct impact on neuromodulation, often resulting in an inflammatory state in the brain. This state can be both detrimental and beneficial to both ongoing and future development, depending on context of current physiology. It is important to mention that damage to intestinal barriers may also result in counter-regulatory measures or signaling. 

In the study by Ballok et al., mice with uncontrolled lupus were seen to have damage to the hippocampus because of gliosis, or enlargement and proliferation of the microglial cells, while immunosuppressed mice had comparatively less degeneration than the uncontrolled mice. This same study also examined the brain of a patient with lupus and found a similar reduction in neuronal density in the hippocampal area [45]. More specifically to microglial cells, a recent review from 2022 described microglial cells as “sensors” for microbiota-derived molecules such as lipopolysaccharide (LPS), short chain fatty acids (SCFA), and tryptophan derivatives. As microglia serve in the role of patrolling brain parenchyma and surveying for signs of injury or infection, it is particularly the acute phase reactants from the microbiome that trigger inflammation [46]. 

The effects of microbiome metabolites on the microenvironment and behavior via microglial cell interactions are not limited to modulation of inflammation. SCFAs (acetate, propionate, and butyrate) are metabolites synthesized from glucose by fecal microbiota through well-established mechanisms [48]. In mice that have experienced thrombotic stroke, those that received supplementation with SCFAs had subsequent improved recovery of motor function of their affected limb compared to those not supplemented. Additional investigation in these mice demonstrated a modulation of CNS lymphocyte and microglial function via the supplementation of SCFAs, leading to post-stroke recovery [49]. 

In humans, the clinical importance is most noted primarily around children with different social structures. The term “sociobiome” refers to the environmental and social factors that influence diet. Interestingly, infants with older siblings have higher counts of facultative anaerobes and lower levels of clostridia spp. and E. Coli [50]. Other social factors, such as income and diet, may also, therefore, play a role clinically; however, given this complexity, there are currently no studies comparing the microbiomes of different social and economic spheres and associated factors, and thus on their downstream impact on microglia. 

## 7. Influence of Maternal Diet during Pregnancy on Microglia and Resulting Effects on Fetal Neurodevelopment

In this paper we have discussed studies that have illustrated the interconnected nature of neurodevelopment and microglial cells as well as how gut microbiome influences those same CNS innate immune cells. In this section we will discuss the influence the prenatal maternal microbiome appears to have on fetal microglia, and thus fetal and perinatal neurodevelopment. Despite the womb being viewed as a sterile environment, it is known that microbiome-derived molecules are able to penetrate the placenta and enter fetal circulation [51]. 

A healthy diet for expecting mothers consists of proteins, carbohydrates, fats, vitamins, minerals, and lots of water. It is understood that the previously listed nutrients are transmitted to the fetus via the placenta. We know that the maternal diet is the primary source of nutrition for the developing fetus, but how much influence does maternal diet have on fetal neurodevelopment as well? In a review paper by CS Rosenfeld 2021, they discuss thoroughly the relationship between maternal diet-induced immune activation throughout gestation and its effect on the formation of neurodevelopmental disorders in offspring [52]. During normal embryonic development, microglia progress through distinct phases of differentiation with differing gene expression and phenology. The lack of maternal microbiomes during these periods of development influences transcriptomes of microglial cells in the fetal brain, particularly *ly86* and *Aoah*, which are a part of a normal response to LPS. This indicates that maternal microbiota may be needed to prepare developing fetal microglia on how to react to postnatally acquired microbiota [53,54]. In a study conducted by Bordeleau et al., they found that a maternal high-fat diet (mHFD) will result in increased maternal immune activation—evidenced by increased circulating levels of interleukin (IL)-6. In addition to this, they found that the morphology of microglia was altered and there were increased microglial interactions with other cells, such as astrocytes, most notably in the hippocampus of mHFD-exposed male offspring. This could have implications for future memory retention or loss. It was also noted there were decreased microglia-associated extracellular space pockets [55]. Extracellular space is important in allowing for long-distance extra-synaptic communication. Therefore, a decrease in space pockets may cause a decrease in the volume or ability of neurotransmitter communication between neurons. In a subsequent study in 2021, Bordeleau et al. found that offspring of mothers with mHFD also had increased density of myelin in the corpus callosum, but with reduced area of cytosolic myelin channels, which are channels within oligodendrocytes hypothesized to serve the metabolic needs of neurons and facilitate efficient neuronal communication [56,57]. This was concurrent with noted reduction in the density of mature lysosomes in the microglia of the corpus callosum, as well as increased synaptic density. Behaviorally, these mice exhibited alteration in social memory and sensorimotor deficits, indicating that mHFD reduced the phagocytic microglial capacity of their offspring and led to dysfunction of normal myelination and behavioral changes [57]. 

In a separate study analyzing the effects of a maternal high-fat diet, Winther et al. analyzed the effects of this type of diet on the offspring’s emotional behavior in adulthood. They found that a high-fat maternal diet led to offspring mice exhibiting a phenotype more prone to anxiety in an elevated plus maze. Along with the behavioral changes, they found significantly higher mRNA levels of hippocampal cytokine TNF-α mRNA as well as monocyte-chemoattractant protein-1(MCP-1), both pro-inflammatory markers. Both of these molecules were found to be associated with this behavior change. MCP-1 is a chemokine that is increased by the stimulation of TNF-a. TNF-a is an activator of immune cells, specifically microglia and astrocytes. As a result of this activation, microglia produce nitric oxide, which results in loss of neurons by diffusion through membranes and intracellular damage. The authors’ results suggest stress-axis pathways in the hippocampus may contribute to the anxiogenic effects noted in offspring of mHFD [58]. 

One study hypothesized that a maternal diet causing obesity will predict offspring peripheral and central inflammatory outcomes. They hypothesized that this is caused by the maternal diet’s effects on maternal adiposity, where increased fat storage results in increased inflammatory markers leading to a maternal inflammatory state. The data they collected suggested that the maternal diet and adiposity directly predict amygdala microglial counts in offspring. Maternal adiposity was found to influence offspring peripheral inflammatory outcomes based on maternal inflammatory state [59,60]. The influence on amygdala counts, could, in theory, significantly influence behavioral outcomes, particularly emotional traits, as seen in the anxiogenic phenotype demonstrated in mHFD by Winther mentioned previously [58]. From a metabolic perspective, a study done with maternal western-high-fat diet fed to baboons, the microbiome was shifted in the high-fat group and was shown to cause epigenetic changes that would predispose offspring to fibrosis patterns, oxidative stress, and non-alcoholic fatty liver disease. This suggests that the offspring of mothers with a high-fat diet are likely to be more at risk of metabolic disease. 

## 8. Conditions Associated with Maternal Diet and Microbiome

Maternal diet and microbiome can have profound lifelong implications on the health outcomes of offspring. These include, but are not limited to, immune system activation, allergen exposure, neurological development, and metabolism. Many studies have examined the association between the microbiome and allergy and asthma incidence. Exposure to environmental antigens has been shown to impact the maternal and fetal microbiome. Gao et al. attributed these associations to the “farm effect,” antibiotic use, dietary fiber, and psychological stress. The “farm effect” refers to consumption of unpasteurized cow’s milk and exposure to other antigens found in agrarian settings. All of these types of exposures alter maternal and fetal microbiomes and subsequent inflammatory responses in the infant’s life [61]. Other studies further examined the role of developing a gut microbiome diversity necessary for a healthy immune system by exposure to microbes through vaginal delivery and breast milk [62]. Wang et al. analyzed a cohort of breast-fed infants and compared fecal samples from those who developed signs of allergies versus those with no signs. The study found that there were different populations of bacteria in the two groups, further linking the maternal microbiome and breast milk microbiota with fetal immune system and allergy development [63].

Perhaps the most consistent takeaway from studies examining any potential conditions associated with maternal diet and microbiome is that the maternal microbiome is critical for modulation of inflammatory markers. Fonseca et al. used mice treated with Lactobacillus johnsonii supplementation to study the effects of microbiome on immune system reaction to respiratory syncytial virus (RSV). Maternal mice given treatment had offspring with fewer inflammatory cytokines and inflammation following RSV infection [64]. Other conditions associated with pro-inflammatory states included sleep deprivation. Mice were again used in a study to explore the effects of sleep. Yao et al. reported that sleep deprivation leads to neurological inflammation and psychological disturbances in mothers. Pro-inflammatory cytokines, such as IL-1β and TNF-α, were found in much higher levels in sleep-deprived maternal offspring [65]. As mentioned earlier, these cytokines result in recruitment of astrocytes when in the CNS.

Many studies have shown a connection between autism spectrum disorder (ASD) and other neurological disorders such as schizophrenia, attention deficit hypersensitivity disorder (ADHD), anxiety, depression, and epilepsy [66,67,68]. One review reported on the connection between gut dysbiosis and chronic inflammatory states that can negatively impact neurodevelopment of the fetus, potentially leading to neuropsychiatric disorders such as ASD, anxiety, and depression [66]. Iannone et al. described how gastrointestinal dysfunction and distress in patients with ASD is quite common. This study also correlated the severity of psychotic symptoms in schizophrenia with the number of modifications to the gut microbiome [67]. Another mouse study mimicked viral infection by injecting mice with an immunostimulant or cytokines. It found that maternal immune activation alters the neuronal gene expression and development in fetuses. Defects in the dopaminergic system follow patterns seen in schizophrenia. The immune activation also impaired the patterns of dendritic spine formation, which altered the excitatory/inhibitory balance of the limbic system, which was associated with similar pathology in patients with ASD [68].

## 9. Conclusions

A number of recent studies have provided evidence that neurodevelopment and neuropathology is profoundly affected by bacterial molecules derived from the gut microbiome. The Gut–Brain axis acts through a myriad of pathways, but the one focused on in this article is that of the CNS-specific phagocytes, microglia. This cell type is vital for synaptic maturation in the developing brain and for monitoring of the extracellular CNS environment; it responds to cytokines released in response to gut microbiomes, such as TNF-a and IL-1, which have been proven to result in conditions such as anxiety and motor dysfunction. Additionally, maternal microbiomes have been shown to increase expression of such cytokines, which then cross the placenta and can cause the same phenomenon but starting prenatally. When microbiomes are restored, often these symptoms or conditions have been shown to resolve. New evidence has demonstrated that this axis is also important prenatally. Maternal microbiome metabolic activity and inflammatory state has the capacity to influence the microglia of developing fetuses, both via priming microglia for future interaction with postnatally acquired commensal bacteria and on influencing microglial transcriptional activity. This may represent an area for the development of interventional strategies to combat maternal gut dysbiosis and thus promote typical neuroarchitecture in the developing fetus and reduce the risk of future neurodevelopmental disorders. Future studies are required to robustly elucidate the interaction between maternal microbiome and the developing fetal innate immune system. Other future directions could include additional controlled mouse model studies involving diets beyond high fat, incorporating different byproducts and biomarkers known to have implications in common neuropathology. Additionally, this work could serve to further emphasize and solidify the importance of diet on global growth and development in pre and postnatal life.

## Data Availability

Not applicable.

## References

[1] Honda K., Littman D.R. (2016). The microbiota in adaptive immune homeostasis and disease. Nature.

[2] Luczynski P., Whelan S.O., O’Sullivan C., Clarke G., Shanahan F., Dinan T.G., Cryan J.F. (2016). Adult microbiota-deficient mice have distinct dendritic morphological changes: Differential effects in the amygdala and hippocampus. Eur. J. Neurosci..

[3] Hooper L.V., Littman D.R., Macpherson A.J. (2012). Interactions between the microbiota and the immune system. Science.

[4] Braniste V., Al-Asmakh M., Kowal C., Anuar F., Abbaspour A., Tóth M., Korecka A., Bakocevic N., Ng L.G., Kundu P. (2014). The gut microbiota influences blood-brain barrier permeability in mice. Sci. Transl. Med..

[5] Nayak D., Roth T.L., McGavern D.B. (2014). Microglia development and function. Annu. Rev. Immunol..

[6] Nayak D., Zinselmeyer B.H., Corps K.N., McGavern D.B. (2012). In vivo dynamics of innate immune sentinels in the CNS. Intravital.

[7] Vaziri Z., Saleki K., Aram C., Alijanizadeh P., Pourahmad R., Azadmehr A., Ziaei N. (2023). Empagliflozin treatment of cardiotoxicity: A comprehensive review of clinical, immunobiological, neuroimmune, and therapeutic implications. Biomed. Pharmacother..

[8] Ginhoux F., Greter M., Leboeuf M., Nandi S., See P., Gokhan S., Mehler M.F., Conway S.J., Ng L.G., Stanley E.R. (2010). Fate mapping analysis reveals that adult microglia derive from primitive macrophages. Science.

[9] Lichanska A.M., Hume D.A. (2000). Origins and functions of phagocytes in the embryo. Exp. Hematol..

[10] Samokhvalov I.M., Samokhvalova N.I., Nishikawa S. (2007). Cell tracing shows the contribution of the yolk sac to adult haematopoiesis. Nature.

[11] Baghdadi M., Umeyama Y., Hama N., Kobayashi T., Han N., Wada H., Seino K.-I. (2018). Interleukin-34, a comprehensive review. J. Leukoc. Biol..

[12] Wei S., Nandi S., Chitu V., Yeung Y.-G., Yu W., Huang M., Williams L.T., Lin H., Stanley E.R. (2010). Functional overlap but differential expression of CSF-1 and IL-34 in their CSF-1 receptor-mediated regulation of myeloid cells. J. Leukoc. Biol..

[13] Garceau V., Balic A., Garcia-Morales C., Sauter K.A., McGrew M.J., Smith J., Vervelde L., Sherman A., Fuller T.E., Oliphant T. (2015). The development and maintenance of the mononuclear phagocyte system of the chick is controlled by signals from the macrophage colony-stimulating factor receptor. BMC Biol..

[14] Mizuno T., Doi Y., Mizoguchi H., Jin S., Noda M., Sonobe Y., Takeuchi H., Suzumura A. (2011). Interleukin-34 selectively enhances the neuroprotective effects of microglia to attenuate oligomeric amyloid-β neurotoxicity. Am. J. Pathol..

[15] Hua J.Y., Smith S.J. (2004). Neural activity and the dynamics of central nervous system development. Nat. Neurosci..

[16] Paolicelli R.C., Bolasco G., Pagani F., Maggi L., Scianni M., Panzanelli P., Giustetto M., Ferreira T.A., Guiducci E., Dumas L. (2011). Synaptic pruning by microglia is necessary for normal brain development. Science.

[17] Hubel D.H., Wiesel T.N. (1970). The period of susceptibility to the physiological effects of unilateral eye closure in kittens. J. Physiol..

[18] Schafer D.P., Lehrman E.K., Kautzman A.G., Koyama R., Mardinly A.R., Yamasaki R., Ransohoff R.M., Greenberg M.E., Barres B.A., Stevens B. (2012). Microglia sculpt postnatal neural circuits in an activity and complement-dependent manner. Neuron.

[19] Vainchtein I.D., Chin G., Cho F.S., Kelley K.W., Miller J.G., Chien E.C., Liddelow S.A., Nguyen P.T., Nakao-Inoue H., Dorman L.C. (2018). Astrocyte-derived interleukin-33 promotes microglial synapse engulfment and neural circuit development. Science.

[20] Battle D.E. (2013). Diagnostic and Statistical Manual of Mental Disorders (DSM). Codas.

[21] Tetreault N.A., Hakeem A.Y., Jiang S., Williams B.A., Allman E., Wold B.J., Allman J.M. (2012). Microglia in the cerebral cortex in autism. J. Autism Dev. Disord..

[22] Vargas D.L., Nascimbene C., Krishnan C., Zimmermann A.W., Pardo C.A. (2005). Neuroglial activtion and neuroinflammation in the brains of patients with autism. Ann. Neurol..

[23] Morgan J.T., Chana G., Pardo C.A., Achim C., Semendeferi K., Buckwalter J., Courchesne E., Everall I.P. (2010). Microglial activation and increased microglial density observed in the dorsolateral prefrontal cortex in autism. Biol. Psychiatry.

[24] Xu Z.X., Kim G.H., Tan J.W., Riso A.E., Sun Y., Xu E.Y., Liao G.-Y., Xu H., Lee S.-H., Do N.-Y. (2020). Elevated protein synthesis in microglia causes autism-like synaptic and behavioral aberrations. Nat. Commun..

[25] Chen Z., Trapp B.D. (2016). Microglia and neuroprotection. J. Neurochem..

[26] Dunn G.A., Mitchell A.J., Selby M., Fair D.A., Gustafsson H.C., Sullivan E.L. (2022). Maternal diet and obesity shape offspring central and peripheral inflammatory outcomes in juvenile non-human primates. Brain Behav. Immun..

[27] Yang I., Corwin E.J., Brennan P.A., Jordan S., Murphy J.R., Dunlop A. (2016). The Infant Microbiome: Implications for Infant Health and Neurocognitive Development. Nurs. Res..

[28] Salminen S., Gibson G.R., McCartney A.L., Isolauri E. (2004). Influence of mode of delivery on gut microbiota composition in seven year old children. Gut.

[29] Aloisio I., Mazzola G., Corvaglia L.T., Tonti G., Faldella G., Biavati B., Di Gioia D. (2014). Influence of intrapartum antibiotic prophylaxis against group B Streptococcus on the early newborn gut composition and evaluation of the anti-Streptococcus activity of Bifidobacterium strains. Appl. Microbiol. Biotechnol..

[30] Stark P.L., Lee A. (1982). The microbial ecology of the large bowel of breast-fed and formula-fed infants during the first year of life. J. Med. Microbiol..

[31] Harmsen H.J., Wildeboer-Veloo A.C., Raangs G.C., Wagendorp A.A., Klijn N., Bindels J.G., Welling G.W. (2000). Analysis of intestinal flora development in breast-fed and formula-fed infants by using molecular identification and detection methods. J. Pediatr. Gastroenterol. Nutr..

[32] Milani C., Duranti S., Bottacini F., Casey E., Turroni F., Mahony J., Belzer C., Palacio S.D., Montes S.A., Mancabelli L. (2017). The First Microbial Colonizers of the Human Gut: Composition, Activities, and Health Implications of the Infant Gut Microbiota. Microbiol. Mol. Biol. Rev..

[33] Butel M.J., Waligora-Dupriet A.J., Wydau-Dematteis S. (2018). The developing gut microbiota and its consequences for health. J. Dev. Orig. Health Dis..

[34] Kim M., Benayoun B.A. (2020). The microbiome: An emerging key player in aging and longevity. Transl. Med. Aging.

[35] Lloyd-Price J., Abu-Ali G., Huttenhower C. (2016). The healthy human microbiome. Genome Med..

[36] Frank D.N., Pace N.R. (2008). Gastrointestinal microbiology enters the metagenomics era. Curr. Opin. Gastroenterol..

[37] Hooper L.V., Wong M.H., Thelin A., Hansson L., Falk P.G., Gordon J.I. (2001). Molecular analysis of commensal host-microbial relationships in the intestine. Science.

[38] Geuking M.B., Cahenzli J., Lawson M.A., Ng D.C.K., Slack E., Hapfelmeier S., McCoy K.D., Macpherson A.J. (2011). Intestinal bacterial colonization induces mutualistic regulatory T cell responses. Immunity.

[39] Donia M.S., Fischbach M.A. (2015). HUMAN MICROBIOTA. Small molecules from the human microbiota. Science.

[40] Rooks M.G., Garrett W.S. (2016). Gut microbiota, metabolites and host immunity. Nat. Rev. Immunol..

[41] Diaz Heijtz R., Wang S., Anuar F., Qian Y., Björkholm B., Samuelsson A., Hibberd M.L., Forssberg H., Pettersson S. (2011). Normal gut microbiota modulates brain development and behavior. Proc. Natl. Acad. Sci. USA.

[42] Charo I.F., Ransohoff R.M. (2006). The many roles of chemokines and chemokine receptors in inflammation. N. Engl. J. Med..

[43] Alleva D.G., Kaser S.B., Beller D.I. (1997). Aberrant cytokine expression and autocrine regulation characterize macrophages from young MRL+/+ and NZB/W F1 lupus-prone mice. J. Immunol..

[44] McHale J.F., Harari O.A., Marshall D., Haskard D.O. (1999). TNF-alpha and IL-1 sequentially induce endothelial ICAM-1 and VCAM-1 expression in MRL/lpr lupus-prone mice. J. Immunol..

[45] Ballok D.A., Woulfe J., Sur M., Cyr M., Sakić B. (2004). Hippocampal damage in mouse and human forms of systemic autoimmune disease. Hippocampus.

[46] D’Alessandro G., Marrocco F., Limatola C. (2022). Microglial cells: Sensors for neuronal activity and microbiota-derived molecules. Front. Immunol..

[47] Zhou R., Qian S., Cho W.C.S., Zhou J., Jin C., Zhong Y., Wang J., Zhang X., Xu Z., Tian M. (2022). Microbiota-microglia connections in age-related cognition decline. Aging Cell..

[48] Miller T.L., Wolin M.J. (1996). Pathways of acetate, propionate, and butyrate formation by the human fecal microbial flora. Appl. Environ. Microbiol..

[49] Sadler R., Cramer J.V., Heindl S., Kostidis S., Betz D., Zuurbier K.R., Northoff B.H., Heijink M., Goldberg M.P., Plautz E.J. (2020). Short-Chain Fatty Acids Improve Poststroke Recovery via Immunological Mechanisms. J. Neurosci..

[50] Yao Y., Cai X., Ye Y., Wang F., Chen F., Zheng C. (2021). The Role of Microbiota in Infant Health: From Early Life to Adulthood. Front. Immunol..

[51] Gomez de Agüero M., Ganal-Vonarburg S.C., Fuhrer T., Rupp S., Uchimura Y., Li H., Steinert A., Heikenwalder M., Hapfelmeier S., Sauer U. (2016). The maternal microbiota drives early postnatal innate immune development. Science.

[52] Rosenfeld C.S. (2021). The placenta-brain-axis. J. Neurosci. Res..

[53] Thion M.S., Low D., Silvin A., Chen J., Grisel P., Schulte-Schrepping J., Blecher R., Ulas T., Squarzoni P., Hoeffel G. (2018). Microbiome Influences Prenatal and Adult Microglia in a Sex-Specific Manner. Cell.

[54] Thion M.S., Garel S. (2017). On place and time: Microglia in embryonic and perinatal brain development. Curr. Opin. Neurobiol..

[55] Bordeleau M., Lacabanne C., Fernández de Cossío L., Vernoux N., Savage J.C., González-Ibáñez F., Tremblay M.-È. (2020). Microglial and peripheral immune priming is partially sexually dimorphic in adolescent mouse offspring exposed to maternal high-fat diet. J. Neuroinflammation.

[56] Snaidero N., Velte C., Myllykoski M., Raasakka A., Ignatev A., Werner H.B., Erwig M.S., Möbius W., Kursula P., Nave K.-A. (2017). Antagonistic Functions of MBP and CNP Establish Cytosolic Channels in CNS Myelin. Cell Rep..

[57] Bordeleau M., Fernández de Cossío L., Lacabanne C., Savage J.C., Vernoux N., Chakravarty M., Tremblay M.-È. (2021). Maternal high-fat diet modifies myelin organization, microglial interactions, and results in social memory and sensorimotor gating deficits in adolescent mouse offspring. Brain Behav. Immun. Health.

[58] Winther G., Elfving B., Müller H.K., Lund S., Wegener G. (2018). Maternal High-fat Diet Programs Offspring Emotional Behavior in Adulthood. Neuroscience.

[59] Maude B., de Cossío Lourdes F., Mallar C.M., Marie-Ève T. (2021). From Maternal Diet to Neurodevelopmental Disorders: A Story of Neuroinflammation. Front. Cell. Neurosci..

[60] Eckburg P.B., Bik E.M., Bernstein C.N., Purdom E., Dethlefsen L., Sargent M., Gill S.R., Nelson K.E., Relman D.A. (2005). Diversity of the human intestinal microbial flora. Science.

[61] Gao Y., Nanan R., Macia L., Tan J., Sominsky L., Quinn T.P., O’Hely M., Ponsonby A.-L., Tang M.L.K., Collier F. (2021). The maternal gut microbiome during pregnancy and offspring allergy and asthma. J. Allergy Clin. Immunol..

[62] Shu S.A., Yuen A.W.T., Woo E., Chu K.-H., Kwan H.-S., Yang G.-X., Yang Y., Leung P.S.C. (2019). Microbiota and Food Allergy. Clin. Rev. Allergy Immunol..

[63] Wang S., Wei Y., Liu L., Li Z. (2022). Association between Breastmilk Microbiota and Food Allergy in Infants. Front. Cell Infect. Microbiol..

[64] Fonseca W., Malinczak C.A., Fujimura K., Li D., McCauley K., Li J., Best S.K.K., Zhu D., Rasky A.J., Johnson C.C. (2021). Maternal gut microbiome regulates immunity to RSV infection in offspring. J. Exp. Med..

[65] Yao Z.Y., Li X.H., Zuo L., Xiong Q., He W.-T., Li D.-X., Dong Z.-F. (2022). Maternal sleep deprivation induces gut microbial dysbiosis and neuroinflammation in offspring rats. Zool. Res..

[66] Edwards S.M., Cunningham S.A., Dunlop A.L., Corwin E.J. (2017). The Maternal Gut Microbiome During Pregnancy. MCN Am. J. Matern. Child Nurs..

[67] Iannone L.F., Preda A., Blottière H.M., Clarke G., Albani D., Belcastro V., Carotenuto M., Cattaneo A., Citraro R., Ferraris C. (2019). Microbiota-gut brain axis involvement in neuropsychiatric disorders. Expert. Rev. Neurother..

[68] Minakova E., Warner B.B. (2018). Maternal immune activation, central nervous system development and behavioral phenotypes. Birth Defects Res..

